# Mediating effects of women’s empowerment on dietary diversity during pregnancy in Central West Ethiopia: A structural equation modelling

**DOI:** 10.1080/16549716.2023.2290303

**Published:** 2023-12-21

**Authors:** Tizita Dengia Etea, Alemayehu Worku Yalew, Mitike Molla Sisay

**Affiliations:** a Department of Public Health, Ambo University, Ambo, Oromia, Ethiopia; b School of Public Health, Addis Ababa University, Addis Ababa, Ethiopia

**Keywords:** Dietary diversity, pregnant women, education, women’s empowerment, SEM, Ethiopia

## Abstract

**Background:**

Considerable proportions of pregnant women consume inadequately diversified diets in Ethiopia. On the other hand, women’s empowerment is identified as a means of achieving maternal nutrition improvement. However, evidence on the relationship between multiple dimensions of women’s empowerment and dietary diversity during pregnancy is limited in Ethiopia.

**Objective:**

This study aimed to assess the mediating effects of women’s empowerment in the pathway between women’s education and dietary diversity during pregnancy in West Shewa zone, Ethiopia.

**Methods:**

A health facility-based cross-sectional study was conducted among 1,383 pregnant women in 2021. Dietary diversity was measured using the minimum dietary diversity for women (MDD-W) tool. Exploratory and confirmatory factor analyses were employed to identify and validate women’s empowerment dimensions. Structural equation modelling (SEM) was used to examine the pathways linking pregnant women’s education and empowerment to dietary diversity during pregnancy.

**Results:**

From the latent dimensions of women’s empowerment produced by factor analyses, pregnant women’s education was directly associated with household decision-making power, psychological and time dimensions. In turn, household decision-making power, psychological and time dimensions were associated with dietary diversity during pregnancy. The direct relationship between pregnant women’s education and dietary diversity was insignificant, but the total indirect effect and total effect were significant. Household decision-making power, psychological and time dimensions were significant mediators in the relationship between pregnant women’s education and dietary diversity. However, economic dimension was related to neither pregnant women’s education nor dietary diversity.

**Conclusion:**

This study highlights pregnant women with better education are more likely to be empowered in household decision-making, psychological and time dimensions; and those empowered pregnant women are more likely to consume more diverse diets, suggesting women’s access to higher education could have a positive indirect effect on consumption of more diverse diets during pregnancy by empowering women in the study area.

## Introduction

Consumption of diverse food can help meet the increased nutrient requirements during pregnancy to support the needs of the pregnant woman, the fetus and for future lactation [1–3]. Pregnant women with inadequate dietary diversity are likely to be consuming less amounts of nutrients than their requirements. The adverse health outcomes of nutritional deficiencies during pregnancy affect both pregnant women and their offspring, including a higher risk of obstructed labour and death due to postpartum haemorrhage [4], preterm birth [5] and low birth weight [5,6]. In developing countries, a large number of pregnant women do not consume adequately diversified diets during their gestational period [7]. Studies in different regions of Ethiopia show that 49.0% to 84.4% of pregnant women consumed inadequately diversified diets [8–13]. These studies indicated that cereal or grain food groups comprised the highest proportion of pregnant women’s diet, whereas meat, egg and dairy products consumption were very low. The pooled prevalence of inadequate dietary diversity among Ethiopian pregnant women is 59% [7], implying the necessity of addressing the problem.

Understanding the importance of the issue, the United Nations put both empowering women and addressing the nutritional needs of pregnant women among its priority agendas of the Sustainable Development Goals (SDGs) [14]. Moreover, it is suggested that maternal nutrition improvement can be achieved through women’s empowerment [15–17]. Women’s empowerment is defined as ‘the process by which women gain greater control over the circumstances of their lives’ [18]. The various dimensions and indicators of women’s empowerment point out that it is reflected in their decision-making power, ability to access and control resources, a sense of self-worth and power to control their own and family lives [19–21]. It is emphasised that resources, agency and achievements should be indivisible in measuring women’s empowerment. Resource is described as ‘preconditions that enhance the ability to exercise choice’, which includes material, human and social resources. Agency is ‘the ability to define one’s goals and act upon them’, which is reflected in the ability to control resources and decisions that affect important life outcomes including decision-making power and psychological empowerment. Achievement is an outcome of choices including well-being outcomes [19].

Although different indicators and dimensions were used to measure women’s empowerment, some of the available studies reported positive and significant associations of women’s empowerment with dietary diversity of reproductive age women [22–26]. Negative [27] and null associations [22] were also found. According to a study in Ethiopia, women’s empowerment which was measured by Women Empowerment in Agricultural Index was positively associated with women’s dietary diversity [28]. Women’s empowerment was also linked to dietary diversity during pregnancy. For instance, a higher level of empowerment was a predictor in the consumption of more diverse diets among pregnant women. Pregnant women who had greater household decision-making power and economic empowerment were more likely to consume more diverse diets than those with low empowerment level [29]. Besides identifying the direct relationships between women’s empowerment and dietary diversity, generating evidence on pathways to dietary diversity through women’s empowerment provides better insight in solving nutrition related problems. In relation to this, a Bangladesh study which examined the mediating effect of women’s agency found that women’s voice (one of the dimensions of agency) significantly mediated the relationship between schooling and dietary diversity among women [22]. However, there are inadequate studies which examined how the multiple dimensions of women’s empowerment may influence dietary diversity during pregnancy in the Ethiopian context.

On the other hand, women’s higher education status has been identified as one of the significant determinants in the consumption of adequately diversified diets among pregnant women in Ethiopia [7,11,13], education can be used to improve the quality of diet among pregnant women. At the same time, existing literatures underline education as pivotal for women’s empowerment [30–32]. Women’s higher education has been linked to higher economic and household decision-making power [33]. It is known to enhance women’s self-esteem, confidence and practice their rights [32,34].

Even if adequate dietary diversity during pregnancy is positively associated with better pregnancy and birth outcomes, poor dietary diversity contribution to maternal undernutrition during pregnancy is a reality in developing countries including Ethiopia [5]. Nonetheless, little is known in Ethiopia about the role the various dimensions of women’s empowerment play in the dietary diversity of pregnant women. Therefore, this study aimed to examine the mechanism linking pregnant women’s education, empowerment and dietary diversity by using structural equation modelling (SEM). It examined the direct relationships of pregnant women’s education and multiple dimensions of women’s empowerment with dietary diversity during pregnancy. It further assessed the mediating effects of women’s empowerment dimensions in the pathway between pregnant women’s education and their dietary diversity in Central West Ethiopia. We hypothesised that pregnant women with higher education would have higher dietary diversity during pregnancy, and women’s empowerment would mediate this relationship.

## Methods

### Study participants, setting and data

Data for this study were obtained from a prospective follow-up study which enrolled 1,453 pregnant women to assess the effect of women’s empowerment on nutritional status and birth weight among pregnant women attending antenatal care (ANC) at public health facilities found in West Shewa zone, Central West Ethiopia. The study participants were pregnant women in their second and third trimester attending ANC follow-up at 12 randomly selected public health facilities located in West Shewa zone. Those pregnant women <18 years old and without partner were excluded from the study interviewer administered, pretested, structured questionnaire was used to collect data on pregnant women’s socio-demographic and obstetric characteristics, empowerment and dietary diversity. The local language, Afan Oromo, was used to collect the data. Data collectors were females who had completed grade 12 and trained on the techniques of data collection. Midwives and/or nurses working at antenatal care service provision unit identified and linked the pregnant women who fulfilled the inclusion criteria to the data collectors. The data were collected from January to August 2021. Details related to the methods of the study are explained by the authors elsewhere [35].

### Sampling procedure

Multistage sampling technique was applied in this study. Seven districts were randomly selected using lottery method from the 22 districts found in West Shewa Zone. Twelve public health centres were proportionally selected using lottery method by taking into account the number of public health centres in each district. Health facilities ANC client registries consisting list of second and third trimester ANC follow-up clients were used as a sampling frame. According to the ANC registry records of the health facilities, the total number of second and third trimester pregnant women who attended ANC was 2,209. Then, the total sample size was distributed proportionally based on the number of second and third trimester pregnant women who received ANC service in the previous 3 months in each health centre (Supplementary Table S1). A systematic random sampling method was used to select the study participants. Data were collected from every second willing pregnant women after they had received ANC services.

### Variables and analytical strategy

SEM was used for this study, which consisted of measurement and structural parts [36]. The endogenous variables were pregnant women’s dietary diversity and women’s empowerment. Exogenous variables included pregnant women’s education, socio-demographic and obstetric characteristics.

## Endogenous variables

### Pregnant women’s dietary diversity

Pregnant women’s dietary diversity was the outcome of this study. It was measured using MDD-W tool [2] to reduce recall bias. Pregnant women were asked to recall and list the foods they consumed in the past 24 h including snacks. In affirmation consumption was ≥15 g, the women were asked if they had eaten at least a spoonful of the food item throughout the day. The foods consumed were categorised into 10 major food groups: 1) Grains, white roots and tubers, and plantains; 2) Pulses (beans, peas and lentils); 3) Nuts and seeds; 4) Dairy; 5) Meat, poultry and fish; 6) Eggs; 7) Dark green leafy vegetables; 8) Other vitamin A-rich fruits and vegetables; 9) Other vegetables; 10) Other fruits.

Dietary diversity was used as a continuous variable in SEM analysis. For descriptive analysis purpose, it was categorised into adequate and inadequate. A score of 1 was given for a particular food category consumed in the past 24 h and 0 if not consumed. A score ≥5 was categorised as adequate and <5 as inadequate dietary diversity [2]. Special occasions such as holidays or fasting days can inflate or deflate dietary diversity scores. In order to minimise their effect, MDD-W was measured twice in nonconsecutive days, i.e. at enrolment and after a month. Then, the average of the two round measurements was used for analysis purposes. Complete data were collected on 1,383 pregnant women.

### Women’s empowerment

Previous works [19–21] have highlighted resources and agency as important women’s empowerment components; and women can be empowered in multiple dimensions including decision-making, economic, psychological and time dimensions. Hence, 22 items were initially included based on these works in order to capture women’s empowerment for this study. These items and others were used to measure women’s empowerment by the authors elsewhere [35]. Six questions were asked to measure the household decision-making power of the women. The women were asked if they participated in decisions regarding what food to buy and consume, food prepared every day, daily needs household purchases, own healthcare, number of children to have and household money use. A score of 1 was given if the woman participated in the decision-making either alone or jointly with her husband/partner, and a score of 0 was assigned if decision was made by her partner or someone else. The six items used to measure economic dimension assessed whether the woman was engaged in activities with cash income, had cash savings of her own, owned any assets that could help generate income, had direct access to household money in her hand to use, involved in decisions on her earning use and had access to money that can be spent freely. Responses for the questions were dichotomised into 1 = Yes and 0 = No. However, the woman was given a score of 1 if she made decisions alone or jointly, while 0 score was given if others made decisions regarding her earning’s use. Five self-esteem and self-confidence related questions were asked to measure psychological dimension. The women were asked if they felt confident in solving problems by themselves, being an important and respected household members. A score of 2 was given for the response of ‘always’ while ‘sometimes’ and ‘not at all’ were given 1 and 0 scores, respectively. Scores of 4, 3, 2, 1 and 0 were given for ‘completely sure’, ‘somewhat sure’, ‘neither sure nor unsure’, ‘somewhat unsure’ and ‘not at all sure’ answers, respectively, to questions asking the confidence level of women in going to health facility and using family planning even if husband/partner objects. Five items were used to assess time dimension. Values of 0, 1 and 2 were assigned if the indicators’ response to the average time spent on housework daily and the actual time spent on housework the previous day were greater than 10 h, between 6 and 10 h and less than 6 h, respectively. The women were also asked if they had time to participate in community activities (1 = Yes, 0 = No). Responses to women’s satisfaction with time availability for leisure activities were summarised into: 1 ‘satisfied’ and 0 ‘unsatisfied’. Higher values of the items’ responses represented a higher empowerment level. Items related to decision-making and psychological dimensions were used to measure agency, whereas resource was measured using economic and time-related items. These dimensions were used as latent constructs. Details are presented under statistical analysis.

## Exogenous variables: independent and control variables

### Independent variables

#### Pregnant women’s education

Completed grade level was used to measure pregnant women’s education. It was treated as a continuous variable.

#### Control variables

Pregnant women’s, household and obstetric characteristics were considered as confounders. The variables were included as control variables because of their potential effect on either or both dietary diversity and women’s empowerment. Age of respondent, parity, occupational status of the respondent and her partner, and educational status of respondent’s partner were measured as continuous variables. Place of residence was dichotomised into urban and rural areas. The respondents were asked if they had access to mass media (electronic media or written material) or not (1 = Yes, 0 = No). Principal component analysis was conducted on the standard demographic and health survey questions used to create household wealth index. The questions were transformed into bivariate variables. Drinking, cooking and washing water sources, toilet facilities, roof and floor materials were categorised into improved and unimproved, while cooking fuel as solid and clean fuels based on demographic and health survey classification [37]. Households were categorised into high, medium and low tertile based on their wealth index score.

### Statistical analysis

First, descriptive analysis was done using STATA version 16.0 (Stata Corporation, College Station, Texas). Next, exploratory and confirmatory factor analyses were conducted to identify the underlying dimensions of women’s empowerment and confirm their generalisability. At last, SEM was used to examine the pathways from pregnant women’s education level to their dietary diversity through multiple dimensions of women’s empowerment. Both factor analyses and SEM were conducted using RStudio version 4.2.3.

Factor analyses were conducted on the 22 items used to measure women’s empowerment since they had not been analysed together priorly. The dataset was divided into two using a random uniform distribution in order to conduct exploratory factor analysis (EFA) with one data sample and confirmatory factor analysis (CFA) with the second data sample. A correlation test was conducted on the items to determine their strength for factor analyses. The Kaiser–Meyer–Olkin test result was 0.77, and the chi-square for the Bartlett test of sphericity was significant at *p* < 0.001. EFA was done to identify the structure and dimensions of women’s empowerment [38]. Weighted least squares estimator was used for EFA, which is appropriate for categorical variables [36,39]. Four factors with eigenvalues >1.0 were produced by EFA and interpreted after oblique (oblimin) rotation. Individual items with a rotated factor loading ≥0.4 were used to construct the different factors [40]. Cross loader item (item loading on two or more factors with pattern coefficient >0.32) [41] and item which contributed for lower reliability coefficient (Cronbach’s alpha <0.7) for a factor were removed step by step. Finally, the four factors were retained based on eigenvalues >1 and interpretability of factors [42,43]. The number of items were reduced to 13.

CFA was done on the four factors identified by the EFA using the second randomly split half data sample to confirm their fitness. Weighted least squares means and variance adjusted (WLSMV) estimator was used, which is the preferred approach for categorical variables analysis [36].

SEM was used to examine the mediating effect of women’s empowerment on dietary diversity during pregnancy. Control variables significant at p-value <0.2 in bivariate analysis were included in the final SEM model as confounders. Estimation for SEM was based on WLSMV, which takes into account categorical variables during analysis [36]. Standardised results were reported.

Bentler comparative fit index (CFI) ≥0.90, Tucker-Lewis index (TLI) ≥0.90, root mean square error of approximation (RMSEA) ≤0.08, and standardised root mean square residual (SRMR) ≤0.1 values were used to determine model fitness for both factor analysis and SEM [40].

## Results

### Descriptive characteristics of study participants

Results of descriptive characteristics of the study participants are presented in Table 1. The mean age of the 1,383 pregnant women was 28.1 years (standard deviation (SD) = 5.3) with a range of 18–44 years. Out of 1,383 pregnant women, the current pregnancy was their first for 586 (42.4%) of them. The mean number of children the pregnant women had was 2.4 (SD = 1.5) with a minimum of 1 and a maximum of 8. The highest and least empowerments for the pregnant women were in household decision-making and psychological dimensions, respectively. Out of 1,383 pregnant women, majority 1,260 (91.1%) of them had involved in decisions on food prepared for the household every day. However, only 550 (39.8%) of them were self-confident to solve problems on their own. About 738 (53.4%) of them spent more than 6 h on housework daily.

**Table 1.** Descriptive characteristics of pregnant women in West Shewa zone, Ethiopia (*n* = 1,383).

Variables	Frequency	Percentage/Mean (SD)
Demographic characteristics		
Women’s education level		
Illiterate	283	20.5
Primary	484	35.0
Secondary and above	616	44.5
Residence		
Rural	531	38.4
Urban	852	61.6
Women’s occupation		
Government employee	290	21.0
Private employee	376	27.2
Farmer	285	20.5
Housewife	402	29.1
Student	30	2.2
Household wealth index		
Low	487	35.2
Middle	446	32.3
High	450	32.5
Women’s empowerment dimensions		
Household decision making power (mean, out of 0–4 total points)		3.6 (1.1)
Economic (mean, out of 0–4 total points)		2.5 (1.4)
Psychological (mean, out of 0–6 total points)		4.3 (1.5)
Time (mean, out of 0–4 total points)		2.9 (1.0)

The mean for the 10 food groups consumed by the pregnant women in the previous 24 h was 4.5 (SD = 0.7) with a range of 2.5 to 6.5 food groups. Of 1,383 pregnant women, about 855 (61.8%) (95% CI: 59.2, 64.4) of them consumed inadequately diversified diets (Table 2).

**Table 2.** Dietary diversity of pregnant women in West Shewa zone, Ethiopia (*n* = 1,383).

Variables	Frequency (n)	Percentage (%)	
Dietary diversity			
Inadequate	855	61.8	
Adequate	528	38.2	
Consumed food groups	First round n (%)	Second round n (%)	Both rounds n (%)
Grains, white roots and tubers, and plantains	1,383 (100)	1,382 (99.9)	1,382 (99.9)
Pulses	1,182 (85.5)	1,110 (80.3)	953 (68.9)
Nuts and seeds	2 (0.1)	22 (1.6)	0 (0.00)
Dairy	586 (42.4)	578 (41.8)	243 (17.6)
Meat, poultry and fish	296 (21.4)	317 (22.9)	80 (5.8)
Eggs	275 (19.9)	312 (22.6)	67 (4.9)
Dark green leafy vegetables	452 (32.7)	463 (33.5)	175 (12.7)
Other vitamin A-rich fruits and vegetables	356 (25.7)	471 (34.1)	113 (8.2)
Other vegetables	1,374 (99.4)	1,300 (94.0)	1,291 (93.4)
Other fruits	273 (19.7)	432 (31.2)	120 (8.7)

### Factor analyses results

Four factors/dimensions were retained based on EFA result. Four items loaded to household decision-making power and economic dimensions; three items loaded to psychological dimension; and two items loaded to time dimensions (Table 3). These factors were modelled as mediator variables in SEM. Reliability (Cronbach’s alpha) test result for household decision-making power, economic, psychological and time dimensions were 0.76, 0.84, 0.74, and 0.81, respectively. Fit indices for the final EFA model were RMSEA = 0.035; TLI = 0.977; and SRMR = 0.02. The correlations among the four factors were significant (*p* < 0.05) and less than 0.42. Fit was acceptable for the final CFA model as well: RMSEA = 0.042; CFI = 0.996; TLI = 0.994; and SRMR = 0.069.

**Table 3.** Factor analyses results of women’s empowerment items in West Shewa zone, Ethiopia, 2021 (*n* = 1,383).

Dimensions	Items	Factor loadings (EFA)	Factor loadings (CFA)
Household decision-making power	Decision on what food to buy and consume (d1)	0.732	0.918
	Decision on food prepared every day (d2)	0.784	0.991
	Decision on own healthcare (d3)	0.663	0.927
	Decision on daily needs household purchases (d4)	0.763	0.943
Economic	Engagement with cash income activities (ec1)	0.891	0.867
	Cash savings of own (ec2)	0.574	0.797
	Decision on own earning use (ec3)	0.692	0.933
	Direct access to household money for use (ec4)	0.591	0.778
Psychological	Self confidence in problem solving (p1)	0.514	0.733
	Feeling as important household member (p2)	0.790	0.933
	Feeling household members respect your opinion (p3)	0.822	0.876
Time	Daily time expenditure on housework (t1)	0.810	0.992
	Time spent on housework the previous day (t2)	0.884	0.847

### SEM results

**Figure 1.**
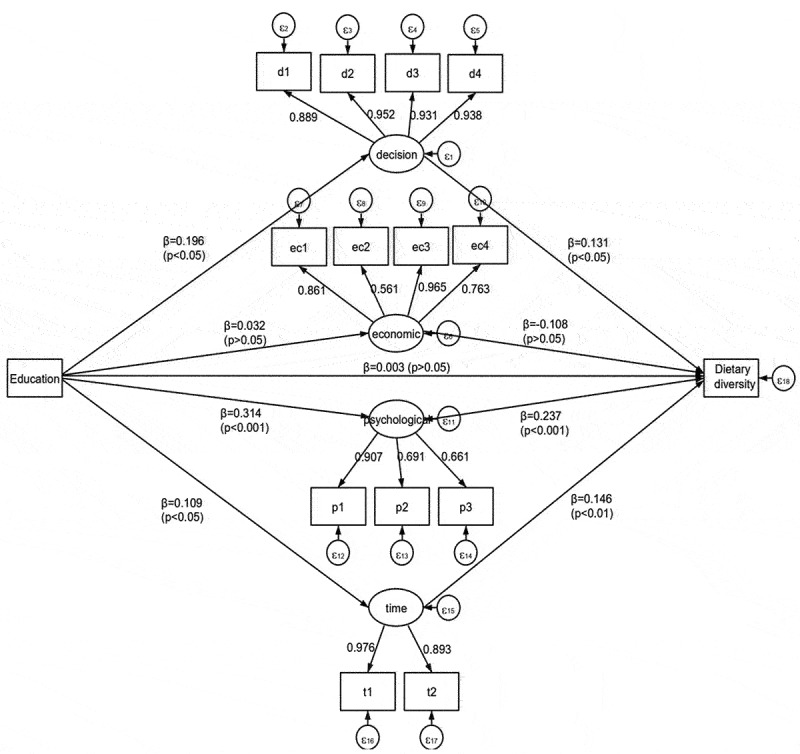
SEM results of the mediation effects of women’s empowerment in the relation between pregnant women’s education and dietary diversity during pregnancy.

Figure 1 shows the adjusted SEM result. The model fit statistics (RMSEA = 0.039, CFI = 0.989, TLI = 0.983, SRMR = 0.072) indicated acceptable fit. Pregnant women’s education level was not directly associated with their economic empowerment dimension (β = 0.032, *p* > 0.05) and dietary diversity during pregnancy (β = 0.003, *p* > 0.05). However, it showed positive direct relations with their household decision-making power (β = 0.196, *p* < 0.05), psychological (β = 0.314, *p* < 0.001) and time (β = 0.109, *p* < 0.05) dimensions. In turn, the three dimensions of pregnant women’s empowerment were directly and positively associated with dietary diversity of pregnant women: household decision-making power (β = 0.131, *p* < 0.05), psychological empowerment (β = 0.237, *p* < 0.001); and time dimension (β = 0.146, *p* < 0.01). Pregnant women’s economic empowerment (β=-0.108, *p* > 0.05) was not associated with their dietary diversity.

The total indirect effect of pregnant women’s education on dietary diversity during pregnancy was positive and significant. Household decision-making power, psychological and time dimensions of women’s empowerment were significant mediators in the relationship between pregnant women’s education and dietary diversity during pregnancy. The total effect of pregnant women’s education on their dietary diversity during pregnancy was also significant (Table 4).

**Table 4.** Direct and indirect effects of pregnant women’s education on dietary diversity during pregnancy in West Shewa zone, Ethiopia (*n* = 1,383).

Effect	Empowerment dimension	Coefficient	*p* value
Total effect		0.115	<0.05
Direct effect		0.003	>0.05
Total indirect effect		0.113	<0.001
Indirect effect through	Household decision making power	0.026	>0.05
	Economic	−0.003	>0.05
	Psychological	0.074	<0.001
	Time	0.016	<0.05

## Discussion

This study used SEM to examine the pathways to dietary diversity during pregnancy in West Shewa zone, Ethiopia. It analysed the direct and indirect effects of women’s educational status on dietary diversity during pregnancy through women’s empowerment dimensions.

Pregnant women’s education was positively associated with their household decision-making power, psychological and time dimensions, but it was insignificantly related with their economic empowerment dimension. Except economic dimension, household decision-making power, psychological and time dimensions were positively associated with dietary diversity of the pregnant women. The direct relation between pregnant women’s education and dietary diversity during pregnancy was insignificant, but it had positive indirect association through mediation effects of household decision-making power, psychological and time dimensions.

The finding of this study showed that about 61.8% of the pregnant women did not consume adequately diversified diets. Other studies in Ethiopia also indicated that more than half of women consumed inadequately diversified diets during their pregnancy [9–13]. It has been pointed out that low dietary diversity may result in micronutrient deficiency among reproductive age women [3,44], which is implicated in negative outcomes for both the mother and fetus [45,46].

The insignificant association of pregnant women’s education status with dietary diversity of pregnant women was unexpected though similar finding was reported from other study in Ethiopia [8]. Better maternal education has been linked with better maternal health and nutrition literacy, and simultaneously converting the acquired knowledge into practice [47,48]. This can be reflected in higher dietary diversity [22]. On the other hand, Ethiopian studies have identified socio-cultural factors such as food taboos, cultural prohibitions to negatively affect dietary practices during pregnancy irrespective of maternal educational status [49,50]. The finding from our study implies the need for further investigations to answer whether education had brought improvements in health and nutrition literacy or not, if there were hindering factors including socio-cultural factors in implementing nutrition knowledge into practice, and whether working outside as educated women had led to double responsibilities forcing less priority to self-care.

In the present study, there was significant association between pregnant women’s education and their household decision-making power. Better education may lead to better household decision-making power [33]. Supporting evidence was found in this study as well. Pregnant women’s decision-making power was also significantly related with their dietary diversity during pregnancy. It is expected that as women’s participation in decisions about food purchase and preparation for household increases, the availability of diverse diet in the household to increase as well; which in turn leads to adequate dietary diversity among the household members including the pregnant women. Majority of the pregnant women were responsible for food management at household level in this study. This may be reflected on dietary diversity of the pregnant women. On the other hand, different factors including nutrition information, socio-cultural factors influence dietary diversity during pregnancy according to Ethiopian studies [7,49,50]. Hence, it is important to understand the mechanisms how these factors influence decision-making power of the pregnant women in relation to the consumption of diverse diets during pregnancy in such culture influenced society. Consistent with this study’s finding, a study in Ghana pointed out women’s decision-making power as a significant contributor to consumption of diverse diets. Women who made decisions regarding household purchases were more likely to eat diverse diet [51]. Yet, Bangladesh study found null association between women’s decision-making power and dietary diversity [22]. Differences in setting and study participants may contribute for varying results. Only pregnant women were included in this study, whereas the referred study was on non-pregnant women.

Pregnant women’s educational status was not associated with their economic empowerment. Furthermore, the direct and indirect null effect of women’s economic empowerment on their dietary diversity was unexpected. It has been highlighted that education enables women to generate their own income by increasing their employment opportunities [32]. In turn, having access and control over economic resources by the women has been linked with consumption of diverse diets [26,52]. The contradicting findings in this study may suggest that (1) pregnant women can involve in income generating activities and have control over resources irrespective of their educational status in the study area; (2) economic empowerment may require the pregnant women work outside for income generation. The tradition role also dictates them to cover household chores. Consequently, the pregnant women may face time poverty for self-care due to heavy workloads. Even if their economic empowerment enables them to afford diverse diets, it may not be transferred to practice. Investigation on the values given for economic empowerment and dietary diversity by the pregnant women may help to better understand the association between them. On the contrary, the finding may suggest that the economic empowerment may not be enough which enables to afford diverse diets rather have the minimum diverse diets per day. The findings may also imply adequate dietary diversity during pregnancy may not necessarily be related with empowerment in every women’s empowerment dimensions. Besides, the interdependences of the empowerment dimensions may have contributed for the insignificant results. Economic empowerment merely may not be a guarantee for adequate dietary diversity among the pregnant women. It may require them simultaneously to be empowered in their either decision-making power or time or psychological dimensions. In order for pregnant women’s economic empowerment to result in adequate diversity during their pregnancy, they may need to involve in decisions regarding food purchase and preparation; have self-worth and the time for self-care.

The significant direct and indirect influence of psychological empowerment on pregnant women’s dietary diversity in this study highlights the crucial role self-confidence and esteem have on consumption of adequately diversified diets during pregnancy. This finding is in-line with the existing evidences that reported significant relations between women’s self-confidence and nutritional status [25,53]. Education enables women to enhance their self-esteem, confidence and practice their rights [32,54]. Supporting result was also found in this study. Such conditions may promote their ability of taking advantage of circumstances and available resources. Therefore, psychologically empowered pregnant women are believed to have the determination to engage in healthy dietary practices including consumption of adequately diversified diets.

This study found out that as education level of the pregnant women increased, the time they spent on housework decreased. Educated women are more likely to be empowered economically [32] because education is believed to increase employment opportunities, thereby decreasing the time women spend on unpaid housework. Working housework for long hours can be tiring. This may in turn reduce the time for self-care. It has been pointed out reducing the time spent on unpaid work enhances women’s empowerment [55]. This study has revealed around 54% of the pregnant women spent between 6 and 16 h daily on housework. Those pregnant women who spent lesser time on housework were more likely to eat adequately diversified diet. The plausible explanation for this may be the pregnant women may be spending more time on income generating activities which enables them afford diverse foods. More importantly, time use has been noted to influence women’s agency [56]. As evidenced in this study as well, educated women are believed to have better decision-making power and self-confidence. Hence, the synergistic interaction of time empowerment with the other empowerment dimensions may have contributed for adequate dietary diversity among the pregnant women empowered in time dimension. Consistent to this study’s finding, reduced working hours among non-pregnant women was significantly associated with their dietary diversity in Nepalese and Ethiopia studies [28,52].

The significant indirect and total effects of pregnant women’s education on their dietary diversity in this study are evidences to the different mechanisms education affects dietary diversity. Pregnant women’s education had indirect significant and positive effect on dietary diversity during pregnancy through pregnant women’s empowerment dimensions. This is an implication that greater dietary diversity among pregnant women may be assured by promoting education which enables empowerment in multiple dimensions. Pregnant women’s education led to adequate dietary diversity among them by empowering their household decision-making ability, psychological and time aspects. As highlighted above, better women’s education may lead to empowerment in decision-making, time and psychological dimensions which may indirectly lead to adequate dietary diversity. A related study in rural Bangladesh indicated that women’s empowerment mediated the significant indirect effect of women’s education on their dietary diversity though different empowerment dimension and non-pregnant women were used [22]. Overall, the significant total effect finding in this study emphasises the crucial role education plays in improving dietary diversity among pregnant women in the study area.

Simultaneous analysis of the different women’s empowerment dimensions on dietary diversity during pregnancy using SEM in this study indicated the differences in the effect of each dimension. The findings suggest intervention programmes which target to improve pregnant women’s dietary diversity in the study area may benefit from strengthening the women’s decision-making power, self-confidence, self-esteem and reducing housework loads. The gender-based division of labour in Ethiopia forces women to spend more time on domestic tasks than men [57] irrespective of women’s employment status, income or education levels [58]. Rural Ethiopian women work up to twice as many hours per day compared to men. Besides the household tasks, they assist their husbands in agricultural activities [59]. However, working with the women’s partners may also help to share household chores responsibilities proportionately between the couples [60]. On the other hand, qualitative study may help explain the contextual nature of the different associations found in this study. Furthermore, it may help to refine the tool used for measuring women’s empowerment by identifying important and unaddressed women’s empowerment items related with pregnant women’s dietary diversity in the study area, and determining the relevance of the indicators included in the study.

This study has some limitations. Self-reported, 24-h recall was used to measure dietary diversity. This may entail social desirability bias as well as it may not reflect the usual consumption. Cross-sectional data were used, so causality could not be inferred. Only partnered pregnant women who were attending antenatal care unit in public health facilities were included in this study. This limits the generalisability of the findings to the excluded segments of women. In Ethiopia, dietary diversity during pregnancy is dependent on multiple factors including nutrition information, dietary diversity knowledge, ANC visits, food security [7,8,11]. Not controlling these factors in this study may be considered as a limitation. Women’s empowerment measuring indicators for this study were developed from existing literatures. There may be missed women’s empowerment aspects relevant to dietary diversity during pregnancy in the study area.

## Conclusion

This study highlights women’s empowerment could be a pathway by which education contributed to adequate dietary diversity during pregnancy in West Shewa zone, Ethiopia. Pregnant women with better education were more likely to be empowered; and those empowered pregnant women were more likely to consume adequately diversified diets. The direct and indirect findings of this study suggest pregnant women’s dietary diversity could be improved through intervention programmes and policies that promote women’s access to better education and women’s empowerment particularly by promoting pregnant women’s household decision-making power, psychological and time empowerment.

## Supplementary Material

Supplementary_Table_S1.docx

## Paper context

Poor dietary diversity affects large proportions of pregnant women. Despite the recognition of women’s empowerment for maternal nutrition improvement, its pathways and effects on dietary diversity during pregnancy are understudied. We observed that pregnant women with better education were more empowered; and those empowered pregnant women were more likely to consume adequately diversified diets. Pregnant women’s household decision-making power, psychological and time empowerment should be considered for improving dietary diversity during pregnancy.
