# Supplementary material for: Mediating effects of women’s empowerment on dietary diversity during pregnancy in Central West Ethiopia: A structural equation modelling
Source: Glob Health Action. 2023 Dec 21;16(1):2290303. doi: 10.1080/16549716.2023.2290303 (PMC10763841; doi:10.1080/16549716.2023.2290303)
Supplement: Supplementary_Table_S1.docx [file ZGHA_A_2290303_SM8532.docx]

Supplementary material

Table S1. Determination of number of study participants based on proportional allocation to size, West Shewa Zone, Central West Ethiopia.

| S.no | District | Randomly selected health centers | No. of 6, 7 and 8 month pregnant women presented at ANC unit in the last 3 months | Allocated sample size ($\frac{n}{N}x\mathrm{ni}$) |
| --- | --- | --- | --- | --- |
| 1 | Ejere | Ejere health center | 157 | 103 |
| 2 | Adda-Berga | Muger health center | 261 | 171 |
| 3 | Adea-Berga | Inchini health center | 159 | 105 |
| 4 | Dendi | Ginchi health center | 316 | 208 |
| 5 | Dendi | Asgori health center | 107 | 70 |
| 6 | Dendi | Faji Gelila health center | 76 | 50 |
| 7 | Dire Inchini | Dire Inchini health center | 219 | 144 |
| 8 | Toke Kutaye | Guder health center | 225 | 148 |
| 9 | Elfeta | Beke health center | 168 | 111 |
| 10 | Elfeta | Gute health center | 119 | 78 |
| 11 | Ambo | Ambo health center | 281 | 185 |
| 12 | Ambo | Awaro health center | 121 | 80 |
| Total sample size | | | | 1,453 |
